# Towards Experiments to Test Violation of the Original Bell Inequality

**DOI:** 10.3390/e20040280

**Published:** 2018-04-13

**Authors:** Andrei Khrennikov, Irina Basieva

**Affiliations:** 1 International Center for Mathematical Modeling in Physics, Engineering, Economics, and Cognitive Science, Linnaeus University, 351 95 Växjö, Sweden; 2 Prokhorov General Physics Institute, Vavilov str. 38D, 119991 Moscow, Russia; 3 Department of Psychology, University of London, London WC1E 7HU, UK

**Keywords:** original Bell inequality, preparation of singlet states, possible experimental test

## Abstract

The aim of this paper is to attract the attention of experimenters to the original Bell (OB) inequality that was shadowed by the common consideration of the Clauser–Horne–Shimony–Holt (CHSH) inequality. There are two reasons to test the OB inequality and not the CHSH inequality. First of all, the OB inequality is a straightforward consequence to the Einstein–Podolsky–Rosen (EPR) argumentation. In addition, only this inequality is directly related to the EPR–Bohr debate. The second distinguishing feature of the OB inequality was emphasized by Itamar Pitowsky. He pointed out that the OB inequality provides a higher degree of violations of classicality than the CHSH inequality. For the CHSH inequality, the fraction of the quantum (Tsirelson) bound $Q_{\mathrm{CHSH}}=2\sqrt{2}$ to the classical bound $C_{\mathrm{CHSH}}=2,$ i.e., $F_{\mathrm{CHSH}}=\frac{Q_{\mathrm{CHSH}}}{C_{\mathrm{CHSH}}}=\sqrt{2}$ is less than the fraction of the quantum bound for the OB inequality $Q_{\mathrm{OB}}=\frac{3}{2}$ to the classical bound $C_{\mathrm{OB}}=1,$ i.e., $F_{\mathrm{OB}}=\frac{Q_{\mathrm{OB}}}{C_{\mathrm{OB}}}=\frac{3}{2}.$ Thus, by violating the OB inequality, it is possible to approach a higher degree of deviation from classicality. The main problem is that the OB inequality is derived under the assumption of perfect (anti-) correlations. However, the last few years have been characterized by the amazing development of quantum technologies. Nowadays, there exist sources producing, with very high probability, the pairs of photons in the singlet state. Moreover, the efficiency of photon detectors was improved tremendously. In any event, one can start by proceeding with the fair sampling assumption. Another possibility is to use the scheme of the Hensen et al. experiment for entangled electrons. Here, the detection efficiency is very high.

## 1. Introduction

In his paper [1] (see also [2]), Bell proposed the probabilistic test based on the EPR-argument [3]. The problem under consideration can be formulated as follows. Einstein, Podolsky, and Rosen proved that quantum mechanics (QM) is incomplete, since its formalism does not represent the EPR elements of reality. Suppose one wants to construct a subquantum theory completing QM. Such a theory should match statistical predictions of QM and, at the same time, *it should describe EPR’s elements of reality.* Can such a theory be local? (as EPR hoped).

Bell proposed a test based on an inequality for correlations. This inequality will be called *the original Bell inequality* (OB inequality). This inequality was proved under the following crucial assumption about coupling the Bell model with hidden variables and the EPR elements of reality.

For the singlet state (as for the original EPR state), spin projections are EPR’s elements of reality. These *elements of reality are equal to measurement outcomes* (elements of reality for $S_{2}$ are measurement outcomes for $S_{1}$). Hence, *values of variables of a subquantum theory beyond the singlet state can be identified with possible outcomes of measurements.* Therefore, for the singlet state, subquantum and quantum correlations can be identified (see Appendix B and Appendix C for further discussion).

However, this beautiful theoretical scheme supporting nonlocal hidden variable theories did not match the experimental framework of that time, since the degree of (anti-)correlations (for the same setting on both sides) was not so high. This problem was solved by transition from the OB inequality to the CHSH inequality [4] or the similar inequalities: the CH74 inequality [5,6] or the Eberhard inequality [7] (see [8] for comparison of these inequalities). Derivations of such inequalities are not based on the assumption of perfect (anti-) correlations. (For convenience, later, we shall compare the OB inequality only with the CHSH inequality, but a similar comparison can be done for other “CHSH-like inequalities” as the CH74 inequality and the Eberhard inequality.) The foundational difference between the OB and CHSH-like inequalities is discussed in Appendix B; see also Appendix C for the general discussion about Bell type inequalities and interpretations of quantum mechanics.

Although the authors think that only a violation of the OB-inequality can be used as the argument in favor of quantum nonlocality, this viewpoint does not match the conventional views. Therefore to stimulate experimenters to perform experiments to violate the OB inequality, we want to highlight that, as was stressed by Itamar Pitowsky [9], the *OB inequality provides a higher degree of violations of classicality than the CHSH inequality.* For the CHSH inequality, the fraction of the quantum (Tsirelson) bound

(1) $$Q_{\mathrm{Tsirelson}}=2\sqrt{2}$$

to the classical bound $C_{\mathrm{CHSH}}=2,$ i.e.,

(2) $$F_{\mathrm{Tsirelson}}=\frac{Q_{\mathrm{CHSH}}}{C_{\mathrm{CHSH}}}=\sqrt{2}$$

is less than the fraction of the quantum bound for the OB inequality

(3) $$Q_{\mathrm{Pitowsky}}=\frac{3}{2}$$

to the classical bound $C_{\mathrm{OB}}=1,$ i.e.,

(4) $$F_{\mathrm{Pitowsky}}=\frac{Q_{\mathrm{OB}}}{C_{\mathrm{OB}}}=\frac{3}{2}.$$

Thus, by violating the OB inequality, it is possible to approach a higher degree of deviation from classicality (see Appendix A for Pitowsky’s comparison of measures of nonclassicality given by quantities $F_{\mathrm{Tsirelson}}$ and $F_{\mathrm{Pitowsky}}$). However, the main message of Pitowsky was not just that $F_{\mathrm{Pitowsky}}>F_{\mathrm{Tsirelson}}$, but that, for multi-dimensional generalizations of the OB inequality,

$$F_{\mathrm{Pitowsky}}\to \infty .$$

(However, for multi-dimensional generalizations of the CHSH-like inequalities, $F_{\mathrm{Tsirelson}}=K_{G}\left(n\right),$ where $K_{G}\left(n\right)$ is the Grothendieck constant of the order n, and, as was shown by Grothendieck, there exists

$$\lim_{n\to \infty }K_{G}\left(n\right)=K_{G},$$

the *Grothendieck constant,* see Appendix A).

Thus, by appealing to multi-dimensional analogs of the OB inequality, experimenters can, in principle, approach an arbitrary large value of the “quantum/classical fraction”.

The main problem for performing an experimental test is that the OB inequality is derived under the assumption of perfect (anti-) correlations. Therefore, it was impossible to perform experiments to check violation of the OB inequality. However, the last few years were characterized by the amazing development of quantum technologies. Technological improvements led to the loophole free tests of the Bell-type inequalities (It may be interesting for the reader that the weblinks to the video-records of the talks of the leaders of all these experimental groups accompanied with the talks of Gregor Weihs and two talks of Philippe Grangier (at the special session BIG EVENT: Final Bells test, at the conference Quantum and Beyond, Växjö, Sweden, June 2016) can be found at the webpage of one of the authors of this paper: https://lnu.se/en/staff/andrei.khrennikov/) (the CHSH, Eberhard, and Clauser–Horne inequalities) [10,11,12] (see also [13,14,15,16,17,18] for previous steps towards these long-aspired experiments) (As was expected by Bell, these experiments did not change the views of those who did not accept the conventional interpretation of experimental outputs, see, e.g., [19]).

One possibility to test violation of the OB inequality is to follow the quantum optics path initiated by Aspect [14]. Nowadays, there exist sources producing with very high probability the pairs of photons in the singlet state. Moreover, the efficiency of photon detectors was improved tremendously. Therefore, one can hope to violate the OB inequality, although this is still the real challenge (see Section 6). In any event, one can proceed under the *fair sampling assumption,* i.e., to solve first the problem of (anti-) correlations.

Another possibility is to test the OB inequality by using the scheme of the Hensen et al. experiment [10]. This experimental scheme does not suffer from inefficiency of detection. However, it seems that the quality of preparation of the singlet state is still insufficient to perform the experimental test to violate the OB inequality (see Section 6).

This paper is a short review based on the results of Pitowsky [9], Ryff [20], and Larsson [21]. Its aim is to collect these results in one text and consider experimental consequences of a combination of the results from Ryff [20] and Larsson [21] in the light of recent tremendous achievements of modern quantum information technologies.

In Section 3, we present probabilistic calculations to estimate the probability of preparation of the singlet state that is sufficient to test violation of the OB-inequality under the assumption of 100% of the detection efficiency. Theorem 2 implies that experimenters have to be able to prepare an ensemble in which more than 75% of pairs are in the singlet state (see also Ryff’s paper [20]). Thus, the existing photon sources of high quality provide the possibility to test the OB inequality, at least under the assumption of fair sampling. In Section 4, we present probabilistic calculations to estimate the minimal efficiency of detection that is sufficient to test violation of the OB-inequality under the assumption of 100% fidelity in preparation of the singlet state. By Theorem 3, the efficiency of the joint detection should be higher than 88,9% (see Larsson’s paper [21] for the original derivation of this bound). In addition, finally, in Section 5, we combined the results of Section 3 and Section 4. By combining 98% level of anti-correlations with 90% level of detection efficiency, one can test violation of the OB inequality.

We remark that generalized (perturbed) Bell’s inequalities that are similar to inequalities obtained in Theorems 2–4 were actively used by one of the coauthors in foundational studies [22,23,24,25].

Successful experimental testing of violation of the OB inequality would be an important (although very challenging) contribution to clarification of quantum foundations.

## 2. Classical and Quantum Bounds for the Original Bell Inequality

We proceed in accordance with Bell’s paper [1]. Let *p* be a probability measure on the space of hidden variables Λ. (Bell used the symbol ρ.) We model measurements on a pair of systems $S_{1}$ and $S_{2}$ with the aid of random variables $A_{s}\left(\lambda \right)$ and $B_{s}\left(\lambda \right),$ where the parameter *s* labels settings of measurement devices, s=a,b,c.

Consider correlations of these random variables given by the integrals:

(5) $$P\left(a,b\right)=\int_{\Lambda }A_{a}\left(\lambda \right)B_{b}\left(\lambda \right)dp\left(\lambda \right).$$

It is assumed that these random variables take values ±1 and that the random variables corresponding to measurements on $S_{1}$ and $S_{2}$ are anti-correlated:

(6) $$P\left(a,a\right)=\int_{\Lambda }A_{a}\left(\lambda \right)B_{a}\left(\lambda \right)dp\left(\lambda \right)=-1.$$

Under these assumptions, Bell derived [1,2] the following inequality:

(7) |P(a,b)−P(a,c)|−P(b,c)≤1

(see also Section 3 for details). We call it the *original Bell inequality* or OB inequality.

This hidden variable model was confronted with spin measurements represented in QM by the spin operators σ·s. In this case, *s* is the unit vector in $R^{3}$ representing the axis of spin projection. Thus, pairwise correlations for spin operators are compared with correlations for random variables. To distinguish measurements on systems $S_{1}$ and $S_{2},$ we shall use symbols $\sigma_{1}\cdot a$ and $\sigma_{2}\cdot b.$

The OB inequality implies that, for classical correlations, the upper bound $C_{\mathrm{OB}}$ for the expression Δ=|P(a,b)−P(a,c)|−P(b,c) equals one. Now, consider the the quantum case. To get perfect anti-correlations, we proceed with the singlet state

(8) $$\Psi =(|+-\rangle -|-+\rangle )/\sqrt{2}.$$

For this state, we have

(9) $$P_{Q}\left(a,b\right)=\langle \sigma_{1}\cdot a⊗\sigma_{2}\cdot b\rangle =-\langle a|b\rangle .$$

One can find the quantum bound for the expression

$$\Delta_{Q}\left(a,b,c\right)=|P_{Q}\left(a,b\right)-P_{Q}\left(a,c\right)|-P_{Q}\left(b,c\right)=\left|\langle a|b\rangle -\langle a|c\rangle \right|+\langle b|c\rangle .$$

**Theorem** **1.** $Q_{\mathrm{OB}}=\max_{a,b,c}\Delta_{Q}\left(a,b,c\right)=\frac{3}{2}.$

**Proof.**  Under the suitable parametrization $\Delta_{Q}\left(a,b,c\right)$ can be represented as

(10) $$\Delta_{Q}\left(\phi_{1},\phi_{2},\theta \right)=2\left|\sin\phi_{1}\sin\phi_{2}\sin\theta \right|+1-2\sin^{2}\phi_{1}.$$

It is easy to find that the maximal value of this function equals to 3/2. ☐

Consider, for example, three vectors in the same plane, $a=\left(1,0\right),b=\left(1/2,-\sqrt{3}/2\right),$$c=(-1/2,-\sqrt{3}/2).$ Then, P(a,b)=−〈a|b〉=−1/2,P(a,c)=−〈a|c〉=1/2,P(b,c)=−〈b|c〉=−1/2. Hence, $\Delta_{Q}\left(a,b,c\right)=3/2.$

Hence, we proved the equality (4), $F_{\mathrm{OB}}=3/2.$

Itamar Pitowsky [9] presented the same argument by using a slight modification of the OB inequality (7).

## 3. Original Bell Inequality: Taking into Account Imperfection of Anti-Correlations

Here, we proceed in Bell’s framework based on classical probability under the assumption that the random variables corresponding to measurements on $S_{1}$ and $S_{2}$ are anti-correlated. As Bell pointed out, this is possible only if the following equality holds

(11) $$A_{a}\left(\lambda \right)=-B_{a}\left(\lambda \right),$$

except a set of measure zero. Bell derived inequality (7) under this assumption of perfect (up to measure zero) anti-correlation. It is easy to modify this equality under assumption of imperfect anti-correlations. Here, we follow the original paper [20], but we proceed in measure theoretic framework. Using the frequentist approach (as in paper [20]) has been objected to by a few authors (see, e.g., [22]).

Suppose that, for each a, there exists a subset $\Lambda_{a}$ of Λ such that Equation (11) holds for all λ from $\Lambda_{a}$ and, for the set $\Lambda_{a}^{\prime }=\Lambda \backslash \Lambda_{a},$ we have:

(12) $$p(\Lambda_{a}^{\prime })\leq \epsilon .$$

Since random variables are dichotomous, on the set $\Lambda_{a}^{\prime }$

(13) $$A_{a}\left(\lambda \right)=B_{a}\left(\lambda \right).$$

Now, on $\Lambda_{b}$, we have:

$$A_{a}\left(\lambda \right)B_{c}\left(\lambda \right)-A_{a}\left(\lambda \right)B_{b}\left(\lambda \right)=A_{a}\left(\lambda \right)A_{b}\left(\lambda \right)A_{b}\left(\lambda \right)B_{c}\left(\lambda \right)+A_{a}\left(\lambda \right)A_{b}\left(\lambda \right)$$

$$=A_{a}\left(\lambda \right)A_{b}\left(\lambda \right)\left[1+A_{b}\left(\lambda \right)B_{c}\left(\lambda \right)\right].$$

On $\Lambda_{b}^{\prime }$, we have:

$$A_{a}\left(\lambda \right)B_{c}\left(\lambda \right)-A_{a}\left(\lambda \right)B_{b}\left(\lambda \right)=$$

$$A_{a}\left(\lambda \right)A_{b}\left(\lambda \right)A_{b}\left(\lambda \right)B_{c}\left(\lambda \right)+A_{a}\left(\lambda \right)A_{b}\left(\lambda \right)-2A_{a}\left(\lambda \right)A_{b}\left(\lambda \right)$$

$$=A_{a}\left(\lambda \right)A_{b}\left(\lambda \right)\left[1+A_{b}\left(\lambda \right)B_{c}\left(\lambda \right)\right]-2A_{a}\left(\lambda \right)A_{b}\left(\lambda \right).$$

Thus,

$$P\left(a,c\right)-P\left(a,b\right)=\int_{\Lambda }A_{a}\left(\lambda \right)A_{b}\left(\lambda \right)\left[1+A_{b}\left(\lambda \right)B_{c}\left(\lambda \right)\right]dp\left(\lambda \right)-2\int_{\Lambda_{b}^{\prime }}A_{a}\left(\lambda \right)B_{b}\left(\lambda \right)dp\left(\lambda \right).$$

Hence, we proved the following theorem:

**Theorem** **2** (Ryff [20])**.** *(Generalization of the OB inequality for imperfect anti-correlations). Under assumption (12), the following inequality for classical correlations holds:*

(14) |P(a,b)−P(a,c)|−P(b,c)≤1+2ϵ.

By introducing the parameter γ=1−ϵ, we write (15) as

(15) |P(a,b)−P(a,c)|−P(b,c)≤3−2γ.

By Theorem 1, we have the inequality: 3−2γ<1,5, i.e., γ>3/4=0,75. Thus, to be able to properly test the OB inequality, one has to be able to produce an ensemble of pairs of quantum systems in which the percentage of precisely (anti-) correlated pairs will be higher than

(16) γ=75%.

## 4. Original Bell Inequality: Taking into Account the Detection Efficiency

For the OB inequality, the issue of the detection efficiency was studied in detail by J.-A. Larsson [21]. Here, we present similar consideration, but in slightly different form, which is consistent with the above presentation of the role of imperfection of correlations. Denote the set of hidden variables for which the pair $A_{a}\left(\lambda \right),B_{b}\left(\lambda \right)$ is detected by the symbol $\Gamma_{ab}.$ The main parameter of the experimental interest is the probability of joint detection of a pair, $p(\Gamma_{ab}).$ For simplicity of considerations, we assume that this probability does not depend on the pair of settings, i.e.,

(17) $$\eta \equiv p(\Gamma_{ab}).$$

Then, correlation conditioned on the pairwise detection is given by

(18) $$\tilde{P}\left(a,b\right)=\frac{1}{\eta }\int_{\Gamma_{ab}}A_{a}\left(\lambda \right)B_{b}\left(\lambda \right)dp\left(\lambda \right).$$

**Theorem** **3.** *Under the assumptions of 100% perfect anti-correlations and set-independent joint detection efficiency (see (17)), the following OB inequality for detectable correlations holds:*

(19) $$|\tilde{P}\left(a,b\right)-\tilde{P}\left(b,c\right)|-\tilde{P}\left(a,c\right)\leq \frac{\left(4-3\eta \right)}{\eta }.$$

**Proof.**  From (18), we get: $\eta \tilde{P}\left(a,b\right)=P\left(a,b\right)-u_{ab},$ where

$$u_{ab}=\int_{\Lambda \backslash \Gamma_{ab}}A_{a}\left(\lambda \right)B_{b}\left(\lambda \right)dp\left(\lambda \right)$$

and, hence, $|u_{ab}|((1-\eta ).$ We have

$$\eta \left(|\tilde{P}\left(a,b\right)-\tilde{P}\left(b,c\right)|-\tilde{P}\left(a,c\right)\right)$$

≤|P(a,b)−P(b,c)|−P(b,c)+3(1−η)≤1+3(1−η).

By dividing both sides of this inequality by η, we obtain (19). ☐

To be able to violate inequality (19), the experimenter has to have sufficiently high the detection efficiency, such that $\frac{\left(4-3\eta \right)}{\eta }<\frac{3}{2},$ i.e., η>8/9=0.889. Thus, the *efficiency of the joint detection should be higher than 88.9%.* This result coincides with the corresponding result from ([21], p. 57). Thus, the detection efficiency should be higher than in the experimental tests for the CHSH inequality [21,26].

## 5. Original Bell Inequality: Taking into Account Imperfection of Anti-Correlations and the Detection Efficiency

**Theorem** **4.** *Under the assumptions (17) and (12), the following experimentally testable version of the OB inequality holds:*

(20) $$|\tilde{P}\left(a,b\right)-\tilde{P}\left(b,c\right)|-\tilde{P}\left(a,c\right)\leq \frac{\left(4+2\epsilon -3\eta \right)}{\eta }.$$

**Proof.**  We have

$$\eta \left(|\tilde{P}\left(a,b\right)-\tilde{P}\left(b,c\right)|-\tilde{P}\left(a,c\right)\right)\leq \left|P\left(a,b\right)-P\left(b,c\right)\right|-P\left(b,c\right)+3\left(1-\eta \right)$$

≤1+2ϵ+3(1−η).

 ☐

To be able to violate inequality (20), experimenter has to have sufficiently high anti-correlations and the detection efficiency, such that $\frac{4+2\epsilon -3\eta }{\eta }<\frac{3}{2}.$ It is convenient to introduce a new parameter κ=1−ϵ. Then, the generalized OB inequality has the form:

(21) $$|\tilde{P}\left(a,b\right)-\tilde{P}\left(b,c\right)|-\tilde{P}\left(a,c\right)\leq \frac{\left(6-2\gamma -3\eta \right)}{\eta }$$

and the condition for possible violation can written as

(22) 4γ+9η>12.

For example, let γ=0.98. Then, η>0.9. Thus, by approaching the 98% level of anti-correlations and the 90% level of detection efficiency, the experimenter can test the OB inequality.

## 6. Conclusions

The modern quantum technology provides the sources producing photons in the singlet state with very high probability, up to 98% of generated ensemble of pairs. From this viewpoint, it is promising to perform the experimental test for the OB inequality by using entangled photons, cf. with experiments [11,12] to violate the CHSH-like inequalities (the Eberhard and CH inequalities). However, as we have seen, tests for the OB inequality demand higher detection efficiency than tests for the CHSH inequality. We remind readers (see also [8] for discussion) that the detection efficiency is not reduced to the efficiency of photo-detectors. Although nowadays there are available photo-detectors having close to 100% efficiency, this does not solve the problem of the detection efficiency. A weak element of the experimental setup based on quantum optics is a polarization beam splitter, where one can lose 8–13% of photons. This loss can play a crucial role in attempts to lift the detection efficiency from 83% [21,26] in the tests for the CHSH inequality to approximately 90% in the planned experimental test for the OB inequality.

It may be reasonable to proceed under the assumption of *fair sampling*. In addition, such a project seems to be realizable.

If one wants to proceed without the fair sampling assumption, then it is very promising to test violation of the OB inequality by using entangled electron spins, i.e., the scheme of the Hensen et. al. [10] experiment that was done for the CHSH inequality. As was reported in [10], the parameter γ in inequality (21) can be selected as γ≈0.92. It exceeds the bound γ=0.75 (see Equation (16)). Therefore, it seems that such an experiment can already be performed today. 

## Appendix A

Here, we follow the paper of Pitowsky [9]:

We recall that the *Grothendieck constant of the order* *n* (denoted by $K_{G}\left(n\right))$ is defined as the least real number such that

(A1) $$|\sum_{i=1}^{m}\sum_{j=1}^{m}a_{ij}\langle x_{i}|y_{j}\rangle |\leq K_{G}\left(n\right)\underset{X_{i},Y_{j}=\pm 1}{\sup}\left|\sum_{i=1}^{m}\sum_{j=1}^{m}a_{ij}X_{i}Y_{j}\right|,$$

for every natural number *m* , every choice of real matrix elements $a_{ij},i,j=1,\ldots ,m,$ and every choice of unit vectors $x_{1},\ldots ,x_{m},y_{1},\ldots y_{m}\in R^{n}.$ It was proved by Grothendieck that there exists

(A2) $$K_{G}=\lim_{n\to \infty }K_{G}\left(n\right).$$

Tsirelson proved that $K_{G}\left(2\right)=\sqrt{2}$ if all vectors belong to the same plane; he also connected this result with the CHSH-inequality.

Tsirelson also proved the following theorem connecting the inequality (A1) to quantum theory:

**Theorem** **A1.**
*The following conditions on m×m matrix $\left(r_{ij}\right)$ are equivalent:*

- *There exists a finite-dimensional Hilbert space H and Hermitian operators $A_{1},\ldots ,A_{m}$ and $B_{1},\ldots ,B_{m}$ acting in H and having the spectrum in [−1,+1], and a state W on H⊗H such that $r_{ij}=\mathrm{TrW}\left(A_{i}⊗B_{j}\right).$*
- *There exist unit vectors $x_{1},\ldots ,x_{m},y_{1},\ldots y_{m}\in R^{2m}$ such that $r_{ij}=\langle x_{i}|y_{j}\rangle .$*

Thus, the fraction

(A3) $$F_{\mathrm{Tsirelson}}\equiv \underset{m,a_{ij},x_{i},y_{j}\left(\mathrm{unit}\;\mathrm{vectors}\right)}{\sup}\frac{|\sum_{i=1}^{m}\sum_{j=1}^{m}a_{ij}\langle x_{i}|y_{j}\rangle |}{\sup_{X_{i},Y_{j}=\pm 1}\left|\sum_{i=1}^{m}\sum_{j=1}^{m}a_{ij}X_{i}Y_{j}\right|}$$

can be used as a measure of quantumness (see also [27] and references herein for related studies about the “quantum/classical fraction”.).

However, as was emphasized by Pitowsky [9], for the OB inequality, we can get the higher value for the “quantum/classical fraction” (see Theorem 1). (We remark that Theorem 1 is easily generalized to the Bell state: $\Psi =(|+-\rangle +|-+\rangle )/\sqrt{2}.)$ This simple result stimulated Pitowsky to consider the general scheme for estimation of the “quantum/classical fraction” for perfectly correlated observables.

Define A(n) as the least real number such that

(A4) $$\sum_{1\leq i<j\leq n}a_{ij}\langle x_{i}|x_{j}\rangle \leq A\left(n\right)\underset{X_{i}=\pm 1}{\sup}\sum_{1\leq i<j\leq n}a_{ij}X_{i}X_{j}.$$

In contrast to the sequence of the Grothendieck constants $K_{G}\left(n\right),$ the sequence A(n) is unbounded and A(n)=O(logn). Moreover, this is the best bound, i.e., there exists a positive constant *c* such that

(A5) $$\sum_{1\leq i<j\leq n}a_{ij}\langle x_{i}|x_{j}\rangle >c\log n\underset{X_{i}=\pm 1}{\sup}\sum_{1\leq i<j\leq n}a_{ij}X_{i}X_{j}$$

for all *n* and $a_{ij}.$ Thus, the “quantum/classical fraction” approaches infinity! Finally, to connect this result with quantum physics, Pitowsky pointed to the following result following from Theorem A1:

**Corollary** **A1.**
*Given unit vectors $x_{1},\ldots ,x_{n}\in R^{n},$ there exists a finite-dimensional Hilbert space H and traceless Hermitian operators $A_{1},\ldots ,A_{n}$ and $B_{1},\ldots ,B_{n}$ acting in H and having the spectrum ±1, and a state W on H⊗H such that $\mathrm{Tr}W\left(A_{i}⊗I\right)=0,\mathrm{Tr}W\left(I⊗B_{j}\right)=0$ and $\mathrm{Tr}W\left(A_{i}⊗B_{j}\right)=\langle x_{i}|y_{j}\rangle .$*

We remark that these observables are perfectly correlated in the state W.

By Corollary A1, the fraction

(A6) $$F_{\mathrm{Pitowsky}}\left(n\right)\equiv =\underset{a_{ij},x_{i}\left(\mathrm{unit}\;\mathrm{vectors}\right)}{\sup}\frac{\sum_{1\leq i<j\leq n}a_{ij}\langle x_{i}|x_{j}\rangle }{\sup_{X_{i}=\pm 1}\sum_{1\leq i<j\leq n}a_{ij}X_{i}Y_{j}}$$

can be used used as a measure of “quantumness”.

Thus, by using the multidimensional analogs of the OB inequality, one can approach very high values of the “quantum/classical fraction”. In addition, it is impossible to do this with CHSH-like inequalities.

## Appendix B. From the EPR Argument to the Original Bell Inequality

The original Bell project can be formulated as the following:

- Einstein, Podolsky, and Rosen proved the existence of elements of reality (for the very special state).
- This implies that QM is not complete and it has to be considered as emergent from some theory with hidden variables.
- Einstein, Podolsky, and Rosen expected that such a theory would be local. (They did not construct such a theory, but they dreamed for it.)
- Bell’s message based on violation of the OB inequality by (theoretical) quantum correlations: unfortunately, EPR realism is not compatible with locality.

We cite Bell ([1], p. 195): “Since we can predict in advance the result of measuring any chosen component of $\sigma_{2},$ by previously measuring the same component of $\sigma_{1},$ it follows that the result of any such measurement must actually be predetermined. Since the initial quantum mechanical wave function does not determine the result of an individual measurement, this predetermination implies the possibility of a more complete specification of the state.” Thus, Bell’s study was aimed at checking reazability the EPR project: to construct a subquantum model that would match statistical predictions of QM and at the same time describe the EPR elements of reality.

We remark that one could respond straightforwardly to EPR’s argument by saying that measurement of the system without disturbance is impossible because a faster-than-light signal can move from $S_{1}$ to $S_{2}.$ We remark that, for Einstein, Podolsky, and Rosen as well as Bohr, such explanation was not acceptable. In addition, this is the important point (We can mention Bohr’s response to the EPR paper [28]. However, it seems that Bohr did not understand the EPR argument. In any event his reply does not explain the origin of perfect correlations.).

However, Bell proved (see [1], p. 199): “In a theory in which parameters are added to quantum mechanics to determine the results of individual measurements, without changing the statistical predictions, there must be a mechanism whereby the setting of one measuring device can influence the reading of another instrument, however remote. Moreover, the signal involved must propagate instantaneously, so that such a theory could not be Lorentz invariant.” Thus, he treated violation of the OB inequality as the proof of nonlocality of any theory with hidden variables.

Now, we point to the crucial connection between the EPR argument and the OB inequality. For the singlet state (as for the original EPR state), spin projections are EPR’s elements of reality. These elements per definition are equal to measurement outcomes (elements of reality for $S_{2}$ are measurement outcomes for $S_{1}).$ Hence, values of variables of a subquantum theory beyond the singlet state can be identified with possible outcomes of measurements. Therefore, for the singlet state, subquantum and quantum correlations can be identified.

There are no reasons to assume this for a non-singlet state. Therefore, CHSH-like projects that are not straightforwardly based on the perfect (anti-)correlations can be objected from the viewpoint that there is no reason to identify the values of subquantum and quantum variables and hence subquantum and quantum correlations. Subquantum correlations satisfy CHSH-inequality, but quantum correlations violate it. (In particular, this was the viewpoint of De Broglie [29], see also [30] for details and references.). By rephrasing Bell, we can say “that what is proved, by impossibility proofs, is lack of imagination” of possible couplings beween subquantum and quantum correlations (cf. [31]).

Therefore, it is important to perform experimental tests for the OB inequality. This and only this test would imply that the issue of nonlocality has to be considered seriously.

## Appendix C. Interpretations of Violations of Bell Type Inequalities and Interpretations of Quantum Mechanics

De Broglie’s viewpoint [29] on interrelation between subquantum and quantum correlations (see Appendix B) can be generally formulated in the framework of the ontic-epistemic representation of quantum phenomena (see Atmanspacher and Primas [32]). This is the framework of the two-level description of natural phenomena. Besides an epistemic model representing outputs of measurements, one can consider an ontic model of reality as it is when nobody performs measurements. The quantum model is treated as an epistemic model (one of possible models describing knowledge that can be gained through measurements). Possible models with hidden variables are possible ontic models behind the quantum epistemic model.

We remark that the very common (especially among philosophers) statement that “an ontic model is about reality as it is” has to interpreted with caution. Scientists can speak only about models of reality, typically mathematical models. It may be better to follow Hertz, Boltzmann, and Schrödinger (see [33] and also [30]) and to speak about a theoretical model presenting a consistent picture of natural phenomena and an epistemic model representing the results of measurements. We remark that the quantum model cannot be considered as a theoretical model (in the sense of Hertz, Boltzmann, and Schrödinger), because, in particular, the measurement problem has not yet been solved (cf. with the claim of Allahverdyan, Balian, Nieuwenhuizen [34] that they solved this problem).

In such two-level framework, De Broglie’s position [29] is justified. In general, there is no reason to identify the subquantum correlations with quantum ones, especially for the CHSH-like inequalities. In particular, a theoretical model can be based on the continuous description of the field-type, cf. with “Einstein’s dream” [35]. Subquantum correlations are correlations of such subquantum fields. Such correlations trivially violate Bell’s inequality because the range of values of fields is unbounded. The concrete model of this type, *prequantum classical statistical field theory* (PCSFT), was developed in the series of works [31,36,37,38]. It generates correlations coinciding with the quantum correlations. (Here, a wave function determines the covariance operator of a prequantum random field.) The corresponding epistemic model is the threshold detection model [39].

We stress that generally interpretations of violation of Bell type inequalities are rigidly coupled to interpretations of the quantum mechanics (see, e.g., De Muynck [40,41], Fuchs [42,43], Fuchs and Schack [44], Grangier [45,46], ’t Hooft [47,48], De Raedt et al. [49,50] Long, Qin, Yang et al. [51]), or more generally to interrelations between classical (Kolmogorovean) and quantum probabilities (see, e.g., Accardi [52,53], Ballentine [54,55,56], Khrennikov [22,25,57], Hess and Philipp [58], Hess [59]). Finally, we point to works of Khrennikov [60] and Kupczynski [61].
